# Complete Genome Sequence and Biodegradation Characteristics of Benzoic Acid-Degrading Bacterium *Pseudomonas* sp. SCB32

**DOI:** 10.1155/2020/6146104

**Published:** 2020-07-02

**Authors:** Wei Xiang, Xiaolan Wei, Hui Tang, Liangbo Li, Rongshao Huang

**Affiliations:** ^1^Department of Agronomy, Agricultural College of Guangxi University, Nanning 530004, China; ^2^Guangxi Institute of Botany, Chinese Academy of Sciences, Guilin 541006, China

## Abstract

Allelochemicals are metabolites produced by living organisms that have a detrimental effect on other species when released into the environment. These chemicals play critical roles in the problems associated with crop replanting. Benzoic acid is a representative allelochemical found in root exudates and rhizosphere soil of crops and inhibits crop growth. The bioremediation of allelochemicals by microorganisms is an efficient decontamination process. In this research, a bacterial strain capable of degrading benzoic acid as the sole carbon source was isolated. The genome of the strain was sequenced, and biodegradation characteristics and metabolic mechanisms were examined. Strain SCB32 was identified as *Pseudomonas* sp. based on 16S rRNA gene analysis coupled with physiological and biochemical analyses. The degradation rate of 800 mg L^−1^ benzoic acid by strain SCB32 was greater than 97.0% in 24 h. The complete genome of strain SCB32 was 6.3 Mbp with a GC content of 64.6% and 5960 coding genes. Potential benzoic acid degradation genes were found by comparison to the KEGG database. Some key intermediate metabolites of benzoic acid, such as catechol, were detected by gas chromatography-mass spectrometry. The biodegradation pathway of benzoic acid, the ortho pathway, is proposed for strain SCB32 based on combined data from genome annotation and mass spectrometry. Moreover, the benzoic acid degradation products from strain SCB32 were essentially nontoxic to lettuce seedlings, while seeds in the benzoic acid-treated group showed significant inhibition of germination. This indicates a possible application of strain SCB32 in the bioremediation of benzoic acid contamination in agricultural environments.

## 1. Introduction

Aromatic compounds, widely found in the environment as components of plant materials and as pollutants of anthropogenic sources, are usually toxic to cellular systems and must be removed [1]. Benzoic acid is the simplest aromatic acid and a metabolic intermediate for many aromatic compounds [2–4]. Ecosystem pollution resulting from an increase in benzoic acid or sodium benzoate in soil and water poses a hazard to humans and other organisms in the ecosystem. Benzoic acid is commonly used as a pharmaceutical or as an industrial preservative and can also be used in synthetic fibers, coatings, resins, tobacco, and rubber industries. The European Commission determined the toxicity level of benzoic acid in 2005, and the toxicology and adverse effects of benzoic acid have been widely reviewed [5]. The wide application of benzoic acid has resulted in it becoming a common environmental pollutant. In agriculture, benzoic acid also demands attention. Autotoxicity, a common phenomenon in natural and agroecosystems, occurs when a living or decomposing plant releases toxins into the surrounding environment to inhibit the growth and development of the same species [6]. In crop root exudates or rhizosphere soil, benzoic acid and its derivatives accumulate over years of cultivation. Benzoic acid is considered to be the predominant cause of crop autotoxicity [7–9], leading to nutrient imbalance and microbial dysfunction in the soil, which cause continuous cropping obstacles [10, 11].

Traditionally, crop rotation can solve the problems associated with continuous cropping. However, this method is inefficient, so there is an urgent need to develop alternative sustainable techniques. Microbial degradation of benzoic acid or other allelochemicals in the soil determines plant autotoxicity activity [12, 13]. *Panax notoginseng* (Sanqi) is a valuable medicinal material in China with serious continuous cropping obstacles [14, 15]. Our previous studies have shown that there are more organic acids in rhizosphere soil than in uncultivated soil. Benzoic acid has been found in the root exudates of Sanqi, where it significantly inhibited growth [16]. During our investigations, we found some wild cultivars of Sanqi that have been continuously cultivated for 8-10 years in the same field, and these cultivars grow healthily. Usually, after harvesting Sanqi, it is impossible to continue planting in the same field due to the problems of continuous cropping [15]. We determined that the differences between cultivars and continuous cultivation of Sanqi may be related to rhizosphere microorganisms. Such microorganisms should be highly tolerant of allelochemicals and could be used for biodegradation. Researchers have identified a variety of microorganisms that can degrade benzoic acid, such as *Pseudomonas*, *Syntrophus*, and *Streptomyces* [17, 18]. Although biodegradation is a highly efficient technique, numerous factors affect degradation efficiency, including pH, temperature, and oxygen limitations [19]. In terms of the mechanism of degradation, benzoate biodegradation by *Pseudomonas* has been reported to occur via the ortho cleavage pathway [20] or by involving both the meta and ortho pathways depending on the concentration of benzoic acid [21]. Different key enzymes for various metabolic pathways of benzoic acid, such as 1,2-dioxygenase and 2,3-dioxygenase, have been found in bacteria [22, 23]. Since the sequence of the first bacterial genome (*Haemophilus influenzae* Rd) was completed in 1995 [24], the quantity of microbial genomic data has exploded [25]. In biodegradation studies, complete genome sequences can be used to rapidly interpret strain characteristics and predict degradation mechanisms [26, 27].

While some benzoic acid-degrading bacteria have been discovered, the large number of microbial resources warrants further exploration, and there is merit in studying the application of such microbes in bioremediation. In this study, the benzoic acid-degrading strain *Pseudomonas* sp. SCB32 was isolated from Sanqi continuous cropping soils. The effects of substrate concentration, pH, and temperature on the biodegradation of benzoic acid by strain SCB32 were explored. The complete genome sequence of the strain SCB32 was determined, and degradation products of the strain were analyzed by gas chromatography-mass spectrometry (GC-MS) to determine the intermediate metabolites of benzoic acid. A possible degradation pathway was deduced following the combined analysis of genome annotation and MS data. Moreover, the toxicity of degradation products of benzoic acid was measured to investigate potential applications of the strain. Isolating and identifying an efficient benzoic acid-degrading bacterial strain and exploring the mechanisms of degradation are of particular significance for the agricultural environment, as it potentially provides a new way to alleviate the problems associated with continuous cropping.

## 2. Materials and Methods

### 2.1. Chemicals and Medium

Luria-Bertani medium (LB, pH 7.0) contained peptone 10.0 g L^−1^, yeast extract 5.0 g L^−1^, and NaCl 10.0 g L^−1^. A mineral salt medium (MSM, pH 7.0) contained (NH4)_2_SO_4_ 1.0 g L^−1^, KH_2_PO_4_ 0.5 g L^−1^, K_2_HPO_4_ 1.5 g L^−1^, NaCl 1.0 g L^−1^, and MgSO_4_·7H_2_O 0.1 g L^−1^. Solid medium plates were prepared by adding 18~20 g L^−1^ agar into the abovementioned liquid media. All media were sterilized by autoclaving at 121°C for 30 min. Benzoic acid (99.5% purity) was purchased from Sinopharm Chemical Reagent Beijing Co., Ltd., China. All other chemical reagents were of analytical grade. Ultrapure water (18.2 M*Ω* cm^−1^) was prepared with a YPK-11 water purification system (Youyue, Chengdu, China).

### 2.2. Enrichment and Isolation of Benzoic Acid-Degrading Bacteria

Rhizosphere soil of *Panax notoginseng* was sampled from Baise City (23°34′11^″^N, 105°55′56^″^E), Guangxi Province, China. Five grams of moist soil sample was added to Erlenmeyer flasks containing 100 mL MSM supplemented with 50 mg L^−1^ benzoic acid. Cultures were incubated at 30°C and 180 rpm for 7 days for enrichment. Thereafter, 10 mL of culture was sampled every 7 days and transferred to fresh MSM, in which the concentration of benzoic acid was gradually increased to 100, 150, and 200 mg L^−1^. Bacterial strains that degraded benzoic acid in the culture were isolated and purified following the procedure described by Buermans and den Dunnen [26]. All media, pipette tips, and Erlenmeyer flasks were sterilized by autoclaving at 121°C for 20 min before use.

### 2.3. Evaluation of Ability of Isolated Strains to Degrade Benzoic Acid

A single colony from an agar plate was inoculated into liquid MSM containing 200 mg L^−1^ benzoic acid and was incubated at 30°C and 180 rpm to logarithmic phase. Bacteria were collected and washed with 0.05 M PBS (pH 7.0), then resuspended in the same concentration of PBS to an OD_600_ of 1.0. Five milliliters of this bacterial culture was inoculated into 100 mL MSM containing 200 mg L^−1^ of benzoic acid. After incubation for 48 h at 30°C and 180 rpm, microbial growth was observed and the benzoic acid residue was quickly analyzed using a UV-1800 spectrophotometer (Shimadzu, Japan); the analysis method was performed as described by Mahboubifar et al. [28]. All experiments were performed three times.

### 2.4. Identification of Strain SCB32

Morphological characteristics were determined using an optical microscope (Nikon, Eclipse E100, Japan) after incubation for 24 h at 30°C. Gram staining was performed according to the method described by Claus, and physiological and biochemical identification was accomplished using the VITEK GN test. Total DNA of strain SCB32 was extracted using a Bacterial Genomic DNA Extraction Kit (Sangon, China), and the 16S rRNA gene was amplified from genomic DNA using the universal primers 27F and 1492R [29]. PCR products were purified and sequenced by Sangon Biotech (Shanghai, China). The resulting 16S rRNA gene sequences were submitted to GenBank and BLAST search by the EzBioCloud identification service [30], and average nucleotide identity (ANI) was determined through the TrueBac ID system [31]. The closest reference sequences were then obtained from the GenBank database, and a phylogenetic tree was generated using MEGA 6.1 software with a neighbor-joining algorithm (bootstrap analysis was performed with 1000 replicates).

### 2.5. Biodegradation Characterization of Strain SCB32

The effects of environmental factors on benzoic acid degradation by strain SCB32 were evaluated in a series of batch experiments performed in 500 mL Erlenmeyer flasks. The initial series determined the concentration range, optimum pH, and temperature for benzoic acid degradation by selected strains. Single-factor optimization tests were applied for conditions including concentration ranges (200, 400, 600, 800, 1000, and 1200 mg L^−1^), pH (5.0, 6.0, 7.0, 8.0, and 9.0), and temperature (20, 25, 30, 35, and 40°C); in the pH and temperature tests, the initial concentration of benzoic acid was maintained at 800 mg L^−1^. The second series measured the degradation efficiency and growth of strain SCB32 under optimized conditions (800 mg L^−1^, pH 7.0, and 30°C). The residual concentration and cell density of strain SCB32 were measured every 2 h, and concurrently, another sample was stored at -80°C for analysis of degradation pathways. For all experiments, the preparation method of the test bacterial solution was the same as above, and a culture medium without bacteria was used as a control.

### 2.6. Genome Sequencing and Bioinformatics Analysis

Genomic DNA was extracted using a QIAamp kit (Qiagen, Germany). The quantity of DNA was measured using a NanoDrop 2000 spectrophotometer (Thermo Scientific, USA). The genome of strain SCB32 was sequenced by Single Molecule, Real-Time (SMRT) technology (PacBio) at Novogene (Beijing, China). Low-quality reads were removed by SMRT Link v5.0.1, and the filtered reads were assembled to generate one contig without gaps. The genome was annotated through the databases GO (Gene Ontology) [32], KEGG (Kyoto Encyclopedia of Genes and Genomes) [33], COG (Clusters of Orthologous Groups) [34], NR (Non-Redundant Protein Database databases) [35], and Swiss-Prot protein database [36].

### 2.7. GC-MS Analyses of Benzoic Acid Metabolites

Cocultures of strain SCB32 were grown in 500 mL basal medium with 800 mg L^−1^ benzoic acid to detect metabolites of benzoate metabolism. Samples (50 mL) were withdrawn from the cultures at various times and centrifuged at 9500 rpm and 4°C for 5 min. The resulting supernatant was acidified with 200 *μ*L 12 M HCL and then extracted three times with 25 mL ethyl acetate [21]. Ethyl acetate extracts were dried under nitrogen gas and then derivatized with *N*,*O*-bis(trimethylsilyl)trifluoroacetamide (TMS). One microliter of the derivatized sample was injected into a GC-MS system (Varian 300-MS, USA). The GC conditions were as follows: carrier gas, helium; splitless mode; temperature programming, 50°C (5 min), 50-140°C (25°C min^−1^), and 140-250°C (5°C min^−1^). Separation was achieved on a VF-5ms column (30 mm × 0.25 mm × 0.25 *μ*m). Mass spectra were obtained at an electron impact (EI) of 70 eV.

### 2.8. Bioassay of Benzoic Acid Degradation Products

Seeds of lettuce (*Lactuca sativa* L.) cultivar “916” (Guangxi, China) were used for bioassay of benzoic acid degradation products. Lettuce seeds were sterilized with sodium hypochlorite and placed in a 9 cm diameter Petri dish containing two layers of sterile filter paper. The filter paper was pretreated with 10 mL benzoic acid degradation products (strain SCB32 was inoculated into liquid MSM containing 800 mg L^−1^ benzoic acid for 48 h), MSM with 800 mg L^−1^ benzoic acid, or MSM alone. Thirty seeds were placed in each dish, and each treatment was repeated three times. The seeds were placed in an incubator at 25°C with a 12 h light/12 h dark photoperiod and kept moist during incubation. After 7 days, the germination rate and fresh weight were measured.

### 2.9. Statistical Analyses

All analyses were performed using SPSS v24.0 software. All datasets were subjected to an analysis of variance (ANOVA) and expressed as mean ± SE. Statistical significance of differences (*p* < 0.05) was assessed by Duncan's multiple range test.

## 3. Results

### 3.1. Isolation and Identification of Benzoic Acid-Degrading Strains

**Figure 1:**
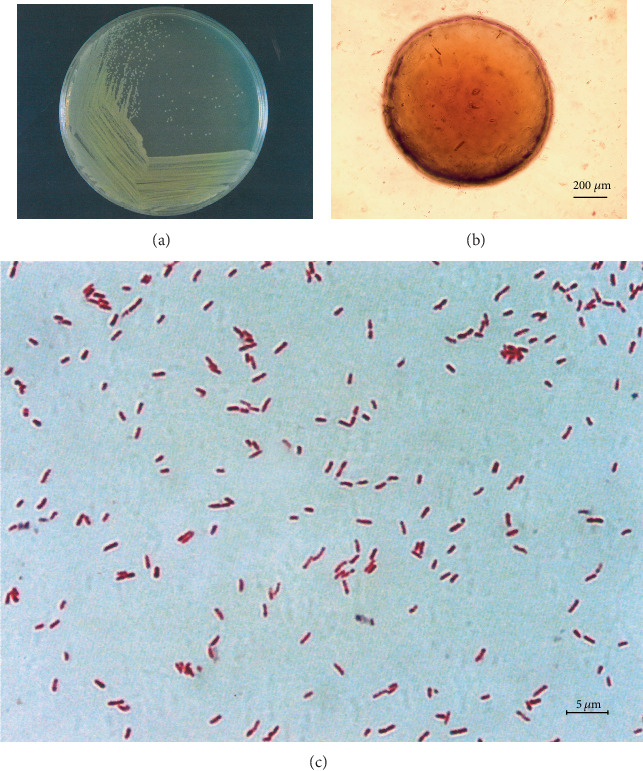
Morphological features of strain SCB32. Colony morphology (a), colony morphology (b), and Gram staining (c).

**Figure 2:**
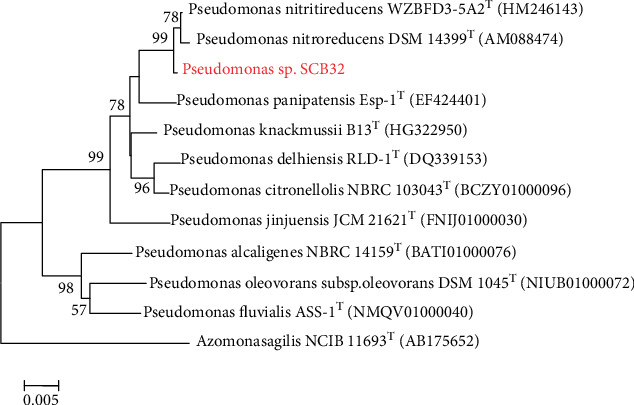
Phylogenetic tree based on 16S rRNA gene sequences showing the relationship between the members of the genus *Pseudomonas*. Bootstrap values (percentages) are based on 1000 replicates and are shown for branches with more than 50% bootstrap support. Superscript “T” indicates a type strain.

After 5 weeks of enrichment, domestication, and separation, four strains of benzoic acid-degrading bacteria were obtained from the soil samples. All four strains could grow in MSM with benzoic acid as the sole carbon source. The isolates exhibited variable degradation capacity (32.90-84.34%) with 200 mg L^−1^ benzoic acid in liquid MSM (data not shown). Strain SCB32 showed the greatest degradation ability and was therefore selected for further study. Cells of strain SCB32 were Gram stain-negative, rod-shaped, and 0.3-0.7 *μ*m × 0.9-2.1 *μ*m and were arranged singly or in pairs. Colonies of the isolate were light yellow, round, convex, and moist with bacterial opacity when cultured on LB agar for 24 h at 30°C (Figure 1). Based on 16S rRNA gene sequence comparison and phylogenetic analysis (Figure 2), strain SCB32 had 99.66% similarity to *Pseudomonas nitritireducens* WZBFD3-5A2^T^. Additionally, ANI analysis showed that the strain was more than 80% similar to the member of the genus *Pseudomonas*. On the basis of these results, the isolate was designated *Pseudomonas* sp. SCB32. VITEK GN test results and ANI data are shown in Supplementary Tables S1 and S2.

### 3.2. Characterization of Benzoic Acid-Degrading Strain SCB32

**Figure 3:**
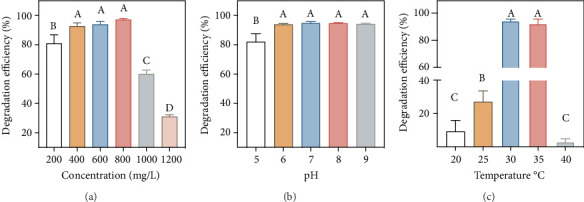
Effects of initial concentration (a), pH (b), and temperature (c) on the degradation of benzoic acid by strain SCB32. Values presented are means. Error bars represent the standard error of three replicates. Identical capital letters in each panel indicate no significant differences between treatments at *p* < 0.05.

Degradation of benzoic acid by strain SCB32 at different initial concentrations, temperatures, and pH is shown in Figure 3. After incubation for 48 h, the degradation rate of benzoic acid at a concentration of 800 mg L^−1^ was greater than 97.0%. However, when the concentration of benzoic acid was further increased to 1000 or 1200 mg L^−1^, the degradation rate dropped significantly (*p* < 0.05) to 31.1% (Figure 3(a)). The effects of temperature and pH were therefore explored using 800 mg L^−1^ benzoic acid. Strain SCB32 could effectively degrade benzoic acid at pH 5.0 to 9.0 (Figure 3(b)), with the degradation rate greater than 80.0% in all cases. The rate of degradation of benzoic acid was highest at pH 7.0, with a mean degradation rate > 94.7%. However, this degradation rate was not significantly different to those obtained at pH 6.0, 8.0, and 9.0 (*p* < 0.05). Temperature had a strong influence on the degradation rate. High (40°C) and low (20°C) temperatures were unfavorable for growth and degradation ability of strain SCB32, resulting in a degradation rate of less than 10.0% (*p* < 0.05). The optimal degradation temperature was 30°C at 800 mg L^−1^ benzoic acid and resulted in a degradation rate > 94.3% (Figure 3(c)).

**Figure 4:**
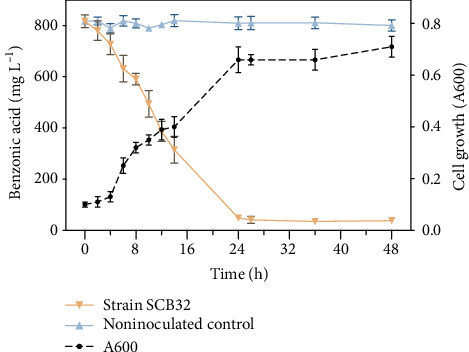
Cell growth and benzoic acid consumption of strain SCB32 in batch culture with initial concentration of 800 mg L^−1^ benzoic acid. Values presented are means. Error bars represent the standard error of three replicates.

Under the optimized conditions (pH 7.0, 30°C, and 800 mg L^−1^ benzoic acid), a negligible acclimation phase was observed (Figure 4). Soon after, cell concentration increased exponentially and benzoic acid was degraded. At 12 h, the benzoic acid had been degraded by more than 50%, and by 24 h, growth of the strain had slowed and the concentration of benzoic acid had dropped to an almost undetectable level.

### 3.3. Genome Sequencing and Annotation

**Figure 5:**
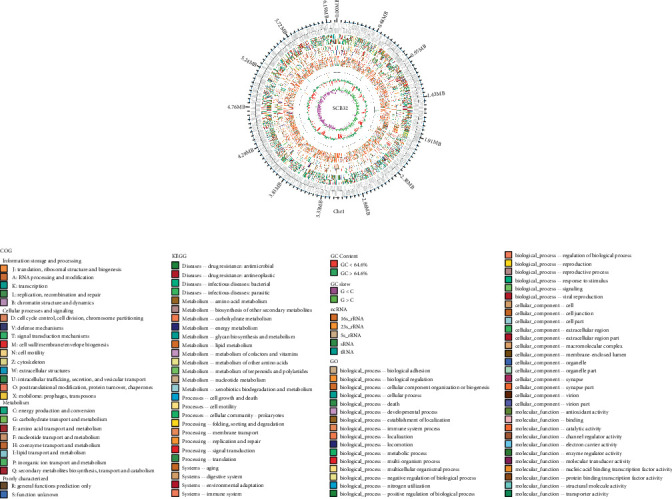
Circular chromosome of *Pseudomonas* sp. SCB32. Circular genome map generated by Circos and showing, from the outside inwards, the following: ring 1, genomic position; ring 2, genes assigned to COG categories; ring 3, genes assigned to KEGG database pathways; ring 4, genes assigned to GO database terms; ring 5, genes assigned to ncRNA; ring 6, GC content; and ring 7, GC skew.

**Figure 6:**
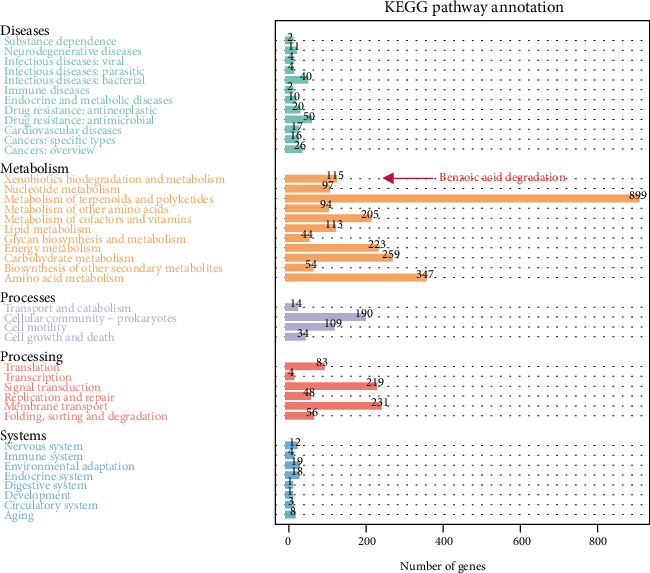
KEGG pathway annotation of genes of strain SCB32.

The extracted total DNA concentration of strain SCB32 was 184 *μ*g *μ*L^−1^, and the OD260/280 value was 1.89, which met the sequencing concentration and quality requirements. The genome sequence of strain SCB32 comprised 6,311,241 bp with a GC content of 64.6% (Table 1). Approximately 5960 coding genes and 71 repeated sequences were predicted. A graphical circular genome map of strain SCB32 is shown in Figure 5. Utilizing COG function assignment, 4503 coding genes could be classified into 25 COG function classes. Cellular components, molecular functions, and biological processes of strain SCB32 were also classified and revealed by genome functional annotation against the GO database. According to KEGG pathway mapping, 90.8% of the total number of coding genes could be assigned to 233 metabolic pathways (Figure 6). Among the predicted genes, 31 were associated with benzoic acid metabolism (Supplementary Table S3).

### 3.4. Identification of Benzoic Acid Metabolites

**Figure 7:**
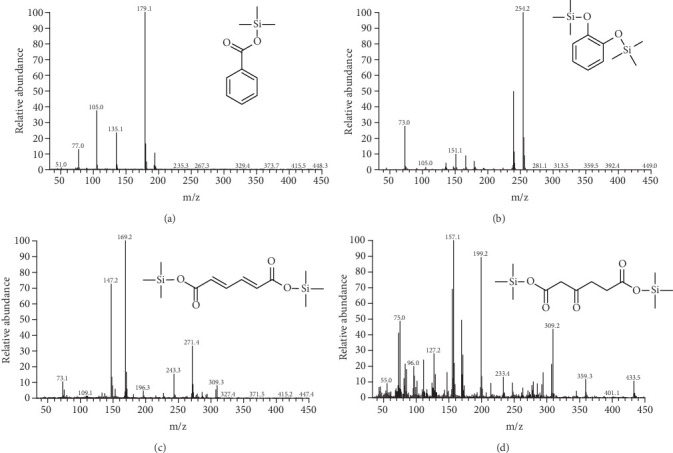
Mass spectra of TMS derivatives of metabolites detected in cultures of benzoic acid-grown strain SCB32. (a) Benzoic acid-TMS, (b) catechol-TMS, (c) *cis*,*cis*-muconic acid-TMS, (d) 3-oxoadipate-TMS.

The benzoic acid degradation process was identified by GC-MS analysis. During the growth of the SCB32 cocultures with benzoate, three TMS derivatives of interest were detected during different stages of the degradation process. The TMS derivative of each compound had the same retention time (RT) and mass spectral profile as the TMS derivative of the National Institute of Standards and Technology (NIST) database. During the 48 h of incubation, catechol-TMS (RT 7.76 min) (Figure 7(b)) was detected first at 4 h. The second compound, *cis*,*cis*-muconic acid-TMS (RT 11.27 min) (Figure 7(c)), was detected at 4, 6, and 8 h. The third compound, at 14 h, was 3-oxoadipate-TMS (RT 9.76 min) (Figure 7(d)). In sterile MSM cultures without inoculation of strain SCB32, benzoic acid changed little and no target metabolites were detected throughout the experiment. These results indicate that benzoic acid degradation by strain SCB32 occurs through the ortho pathway. Benzoic acid may be hydroxylated to form catechol, which is subsequently oxidized by ring fission to yield *cis*,*cis*-muconic acid, followed by muconolactone, and ultimately, downstream products enter the citrate cycle (TCA cycle).

### 3.5. Bioassay of the Benzoic Acid Degradation Products

**Figure 8:**
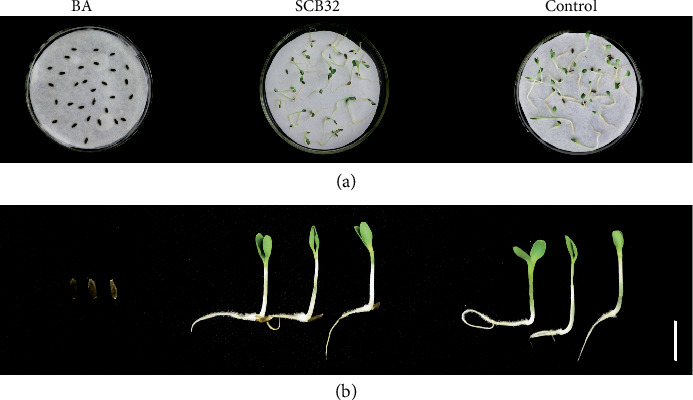
Lettuce seeds exposed to exogenous benzoic acid and its degradation metabolites. Lettuce seeds were treated with MSM (control), 800 mg L^−1^ benzoic acid (BA), or metabolites from BA degraded by SCB32. (a) Lettuce germination and (b) lettuce seeds after 7 d culture. Bar, 1 cm.

**Figure 9:**
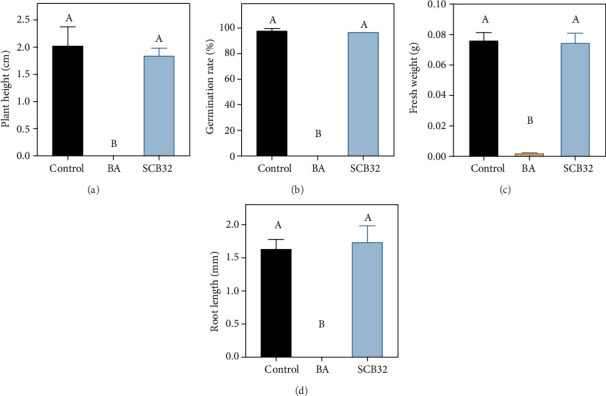
Bioassay on lettuce seeds of benzoic acid and its degradation metabolites produced by strain SCB32. Lettuce seeds were treated with MSM (control), 800 mg L^−1^ benzoic acid (BA), or metabolites from BA degraded by SCB32. Error bars represent the standard error of three replicates. Identical capital letters in each panel indicate no significant differences between treatments at *p* < 0.05.

The toxicity of benzoic acid and its degradation products were evaluated using lettuce seeds (Figures 8 and 9). Benzoic acid at 800 mg L^−1^ had a significant toxic effect on lettuce seeds, with a seed germination rate of 0%. However, when the lettuce seeds were exposed to benzoic acid inoculated with strain SCB32, there was a significant detoxification effect. The degradation products of benzoic acid did not significantly inhibit seed germination rate, plant height, or fresh weight, which were 1.1%, 8.9%, and 2.2% lower than the control (MSM alone), respectively. Interestingly, root length of the degradation product-treated group increased by 6.1% compared with the control.

## 4. Discussion

Allelochemicals, metabolites produced by living organisms that have a detrimental effect on other species when released into the environment, significantly inhibit plant growth [11, 37]. The use of microorganisms to degrade allelochemicals such as benzoic acid and for soil remediation has proven to be effective [38, 39]. In this study, using an enrichment and domestication strategy, a benzoic acid-degrading bacterium, SCB32, was isolated and revealed to be a member of the genus *Pseudomonas*. The genus *Pseudomonas*, belonging to the family *Pseudomonadaceae*, is metabolically diverse and contains more than 190 species with validly published names [40]. The most closely related genus is *Azomonas* [41]. Modern bacterial taxonomy defines species by directly comparing whole genome sequences, and average nucleotide identity (ANI) has been widely recognized as a useful tool in this process [42]. The generally accepted cutoff value for the species boundary is approximately 95~96% ANI [43, 44]. Using the TrueBac ID system [31], the ANI value between strain SCB32 and reference strains was far lower than the cutoff value (Supplementary Table S2). Unfortunately, combined with the identification results of physiological, biochemical, and molecular biology analyses, the specific classification of strain SCB32 cannot currently be determined. A more comprehensive identification of strain SCB32 will be conducted in future work.

Many bacterial species from the genus *Pseudomonas* have been reported to degrade benzoic acid or benzoate, but their degradation ability varies. In this study, when the concentration of benzoic acid was 800 mg L^−1^, strain SCB32 showed the best growth and the highest degradation rate (>97%) in 24 h. But above this concentration, an inhibitory effect was observed. This is because benzoic acid has a known antibacterial effect [5]. Previously reported biodegradable concentrations of benzoic acid were mostly below 500 mg L^−1^. For example, *Pseudomonas* sp. QTF5 can degrade 12.4% of benzoate at a concentration of 3 mg mL^−1^ within 3 days [45]. *Pseudomonas putida* ATCC 49451 degrades approximately 100% of benzoate at a concentration of 400 mg L^−1^ within 12 h. For *P*. *putida* ATCC 49451, substrate inhibition was observed at benzoate concentrations higher than 500 mg L^−1^ [21]. Strain WH-B3 degrades more than 90% of benzoate at a concentration of 500 mg L^−1^ within 12 h [38]. Compared with them, stain SCB32 has a higher degradation capacity. In addition, strain SCB32 shows better pH adaptability for benzoic acid degradation. The benzoic acid degradation rate of strain SCB32 was reduced under specified temperature conditions, determined by the characteristics of the strain itself [29]. The concentrations of benzoic acid in the environment are far lower than the abovementioned tolerable value (800 mg L^−1^), indicating that strain SCB32 has great application potential. Although some environmental pollutants are degraded by microbes, they may produce toxic intermediates such as benzoic acid or catechol and cannot be further degraded [46, 47]. Therefore, autotoxicity of the final degradation products of pollutants is of interest. Lettuce (*Lactuca sativa* L.) is generally accepted as an allelochemical-sensitive plant species, and benzoic acid has a significant toxic effect on it [38, 48]. In this work, benzoic acid (800 mg L^−1^) was degraded by strain SCB32 within 48 h, and the residues of degradation products were phenotypically nontoxic to lettuce seed germination. Strain SCB32 therefore has potential in the biodegradation of benzoic acid for agricultural applications.

**Figure 10:**
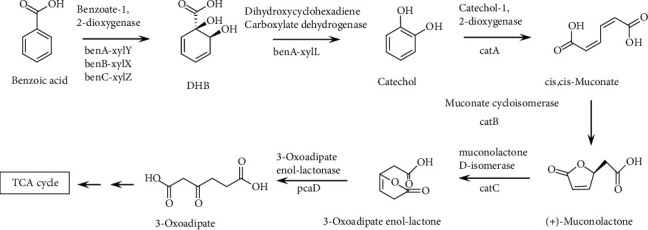
Proposed pathway for benzoic acid metabolism by strain SCB32. Asterisks indicate compounds detected as TMS derivatives in SCB32 coculture extracts.

The metabolic processes of most of the benzoic acid-degrading bacteria are based on presumed evidence, including the identification of metabolites and the detection of key enzyme activities [22, 49], and inferring metabolism is an imperfect task. In contrast, microbial genome analysis and gene annotation allowed us to directly display the various genes responsible for the biodegradation potential of benzoic acid [26], promoting investigation of environmental bioremediation by strain SCB32. In this study, the benzoic acid metabolic pathway based on genome annotation and MS was inferred (Figure 10). Initially, benzoic acid is converted into 1,2-dihydro-1,2-dihydroxybenzoic acid (DHB) by benzoate-1,2-dioxygenase (*benA-xylX*, *benB-xylY*, or *benC-xylZ* gene), then transformed into catechol by the function of the gene *benD-xylL* [50, 51]. Catechol is an intermediate of many aromatic metabolites [52]. Next, catechol, transformed into *cis*,*cis*-muconate, undergoes ring cleavage by catechol-1,2-dioxygenase (*catA* gene) through the ortho pathway [53]. Subsequently, the metabolites of each process undergo a series of transformations under functional genes such as *catB*, *catC*, and *pcaD* and finally degrade to tricarboxylic acid (TCA) cycle [54, 55]. Moreover, using GC-MS analysis, catechol, *cis*,*cis*-muconic acid, and 3-oxoadipate were identified. This is consistent with the pathway based on genome prediction. Some intermediate metabolites were not detected, because they transformed either too fast or at a low level. For example, 1,2-dihydro-1,2-dihydroxybenzoic acid is a very unstable intermediate and is usually consumed rapidly [56]. Comprehensive understanding of the degradation mechanism may help with continued enhancement of the process of bioremediation.

## 5. Conclusions

Data obtained in this study indicate that strain SCB32 could degrade a model allelochemical benzoic acid as the sole carbon source for growth and that it could effectively degrade benzoic acid in MSM. The biodegradation pathway of benzoic acid, the ortho pathway, is proposed for strain SCB32 based on combined data from genome annotation and mass spectrometry. Furthermore, the degradation products of benzoic acid by strain SCB32 had no obvious toxic effect on lettuce germination. Our results provide the groundwork for further elucidation of the genetic basis of benzoic acid degradation in strain SCB32. These results indicate a possible application of strain SCB32 in the bioremediation of benzoic acid contamination in agricultural environments.

## Figures and Tables

**Table 1:** Genome characteristics of strain SCB32.

Characteristic	Value
Genome size (bp)	6,311,241
GC content (%)	64.6
No. of contigs	4
N50 contig length (bp)	6,335,082
No. of genes	5960
Total size of all genes (bp)	5,460,030
Average gene length (bp)	960
No. of tRNA	68
No. of rRNA	19
Genes assigned to COG	4503
Genes assigned to KEGG	5414
Genes assigned to GO	3876
Genes assigned to NR	5493
Genes assigned to Swiss-Prot	2607

## Data Availability

The 16S rRNA gene sequence and genome sequence of strain SCB32 were submitted to the NCBI GenBank database under accession numbers MN559810 and CP045118, respectively.

## Supplementary Materials

Supplementary Materials Table S1: physiological and biochemical characteristic by VITEK GN. Table S2: average nucleotide identity (ANI). Table S3: potential benzoic acid degradation genes found in the isolate SCB322 genome annotated by the KEGG database.
