# Supplementary material for: Complete Genome Sequence and Biodegradation Characteristics of Benzoic Acid-Degrading Bacterium Pseudomonas sp. SCB32
Source: Biomed Res Int. 2020 Jul 2;2020:6146104. doi: 10.1155/2020/6146104 (PMC7354641; doi:10.1155/2020/6146104)
Supplement: Supplementary Materials — Table S1: physiological and biochemical characteristic by VITEK GN. Table S2: average nucleotide identity (ANI). Table S3: potential benzoic acid degradation genes found in the isolate SCB322 genome annotated by the KEGG database. [file 6146104.f1.pdf]

Supplementary Material

Complete Genome Sequence and Biodegradation Characteristics of Benzoic Acid-Degrading Bacterium, *Pseudomonas* sp. SCB32

Wei Xiang [1], Xiaolan Wei [1], Hui Tang [2], Liangbo Li [1,*], Rongshao Huang [1,*]

[1] Department of Agronomy, Agricultural College of Guangxi University; Nanning 530004, China; victorxiang@st.gxu.edu.cn (W.X.); 1917301039@st.gxu.edu.cn (X.W.)

[2] Guangxi Institute of Botany, Chinese Academy of Sciences，Guilin 541006, China; th@gxib.cn（H.T.）

* Correspondence: hrs17252@gxu.edu.cn (R.H); llb100@126.com (L.L.); Tel./Fax: +86-771-323-5612(R.H and L.L.);

Received: date; Accepted: date; Published: date

Table S1. Physiological and Biochemical Characteristic by VITEK GN

VITEK GN							
Ala-Phe-Pro-ARYLAMIDASE	-	ADONITOL	-	L-Pyrrolydonyl-ARYLAMIDASE	-	L-ARABITOL	-
D-CELLOBIOSE	-	BETA-GALACTOSIDASE	-	H_2S PRODUCTION	-	BETA-N-ACETYL-GLUCOSAMINIDASE	-
Glutamyl Arylamidase pNA	-	D-GLUCOSE	-	GAMMA-GLUTAMYL-TRANSFERASE	+	FERMENTATION/ GLUCOSE	-
BETA-GLUCOSIDASE	-	D-MALTOSE	-	D-MANNITOL	-	D-MANNOSE	-
BETA-XYLOSIDASE	-	BETA-Alanine arylamidase pNA	-	L-Proline ARYLAMIDASE	+	LIPASE	-
PALATINOSE	-	Tyrosine ARYLAMIDASE	+	UREASE	-	D-SORBITOL	-
SACCHAROSE/SUCROSE	-	D-TAGATOSE	-	D-TREHALOSE	-	CITRATE(SODIUM)	+
MALONATE	-	5-KETO-D-GLUCONATE	-	L-LACTATE alkalinisation	+	ALPHA-GLUCOSIDASE	-
SUCCINATE alkalinisation	+	Beta-N-ACETYL-GALACTOSAMINIDASE	-	ALPHA-GALACTOSIDASE	-	PHOSPHATASE	-
Glycine ARYLAMIDASE	-	ORNITHINE DECARBOXYLASE	-	LYSINE DECARBOXYLASE	-	L-HISTIDINE assimilation	-
P-COUMALIC ACID	+	BETA-GLUCORONIDASE	-	O/129 RESISTANCE	-	Glu-Gly-Arg-ARYLAMIDASE	-
L-MALATE assimilation	-	ELLMAN	-	L-LACTATE assimilation	+		

+ positive, - negative

Table S2. Average nucleotide identity (ANI).

No.	Hit Taxon	Strain name	ANI (%)	16S rRNA (%)	Taxonomy
1	*Pseudomonas nitritireducens*	WZBFD3-5A2^{T}	89.09	99.66	Pseudomonadales; Pseudomonadaceae; *Pseudomonas*
2	*Pseudomonas nitroreducens*	LMG 21614^{T}	89.04	99.66	Pseudomonadales; Pseudomonadaceae; *Pseudomonas*
3	*CP004143_s* *	ATCC 13867	91.04	99.52	Pseudomonadales; Pseudomonadaceae; *Pseudomonas*
4	*Pseudomonas panipatensis*	LMG 24738^{T}	86.42	98.90	Pseudomonadales; Pseudomonadaceae; *Pseudomonas*
5	*Pseudomonas knackmussii*	B13^{T}	86.83	98.77	Pseudomonadales; Pseudomonadaceae; *Pseudomonas*
6	*Pseudomonas citronellolis*	DSM 50332^{T}	87.16	98.56	Pseudomonadales; Pseudomonadaceae; *Pseudomonas*
7	*Pseudomonas delhiensis*	LMG 24737^{T}	87.15	98.22	Pseudomonadales; Pseudomonadaceae; *Pseudomonas*
8	*Pseudomonas jinjuensis*	JCM 21621^{T}	86.90	98.22	Pseudomonadales; Pseudomonadaceae; *Pseudomonas*
9	*Pseudomonas alcaligenes*	NCTC 10367^{T}	85.11	96.92	Pseudomonadales; Pseudomonadaceae; *Pseudomonas*
10	*Pseudomonas fluvialis*	ASS-1^{T}	84.63	96.71	Pseudomonadales; Pseudomonadaceae; *Pseudomonas*
11	*CP000744_s* *	AZPAE14941	85.71	96.64	Pseudomonadales; Pseudomonadaceae; *Pseudomonas*
12	*Pseudomonas aeruginosa*	DSM 50071^{T}	85.67	96.64	Pseudomonadales; Pseudomonadaceae; *Pseudomonas*
13	*CP015992_s* *	TCU-HL1	85.18	95.88	Pseudomonadales; Pseudomonadaceae; *Pseudomonas*
14	*Pseudomonas furukawaii*	KF707^{T}	85.12	95.74	Pseudomonadales; Pseudomonadaceae; *Pseudomonas*
15	*CP032616_s* *	DY-1	84.89	95.74	Pseudomonadales; Pseudomonadaceae; *Pseudomonas*
16	*JYKO_s* *	LFM046	85.19	95.53	Pseudomonadales; Pseudomonadaceae; *Pseudomonas*
17	*Pseudomonas flexibilis*	ATCC 29606^{T}	84.68	95.34	Pseudomonadales; Pseudomonadaceae; *Pseudomonas*

Superscript "T" indicates a type strain. Superscript "*" indicates a Genomospecies. A genomospecies is a tentatively novel species that is supported by genomic evidence and identified by TrueBac ID [1, 2].

Table S3. Potential benzoic acid degradation genes found in the isolate SCB322 genome annotated by the KEGG database

Gene_id	genes	Enzymes	E.C. number
SCB32_GM003205	*benA-xylX*	benzoate 1,2-dioxygenase subunit alpha	1.14.12.10<br>1.14.12.-
SCB32_GM003204	*benB-xylY*	benzoate 1,2-dioxygenase subunit beta	1.14.12.10<br>1.14.12.-
SCB32_GM003203	*benC-xylZ*	benzoate 1,2-dioxygenase reductase component	1.18.1.-
SCB32_GM003202	*benD-xylL*	dihydroxycyclohexadiene carboxylate dehydrogenase	1.3.1.25 1.3.1.-
SCB32_GM003199	*catA*	catechol 1,2-dioxygenase	1.13.11.1
SCB32_GM003054	*catB*	muconate cycloisomerase	5.5.1.1
SCB32_GM003200	*catC*	muconolactone D-isomerase	5.3.3.4
SCB32_GM002267	*pobA*	p-hydroxybenzoate 3-monooxygenase	1.14.13.2
SCB32_GM002271	*pcaG*	protocatechuate 3,4-dioxygenase, alpha subunit	1.13.11.3
SCB32_GM002270	*pcaH*	protocatechuate 3,4-dioxygenase, beta subunit	1.13.11.3
SCB32_GM003195	*pcaB*	3-carboxy-cis, cis-muconate cycloisomerase	5.5.1.2
SCB32_GM003193	*pcaC*	4-carboxymuconolactone decarboxylase	4.1.1.44
SCB32_GM003194	*pcaD*	3-oxoadipate enol-lactonase	3.1.1.24
SCB32_GM003196	*pcaF*	3-oxoadipyl-CoA thiolase	2.3.1.174
SCB32_GM004010	*mhpD*	2-keto-4-pentenoate hydratase	4.2.1.80

SCB32_GM002434	*bphH, xylJ, tesE*	2-oxopent-4-enoate	4.2.1.80 4.2.1.132
SCB32_GM002436	*bphI, xylK, nahM, tesG*	4-hydroxy-2-oxovalerate	4.1.3.39 4.1.3.43
SCB32_GM002435	*bphJ, xylQ, nahO, tesF*	acetaldehyde/propanal dehydrogenase	1.2.1.10 1.2.1.87
SCB32_GM003470	*praC, xylH*	4-oxalocrotonate tautomerase	5.3.2.6
SCB32_GM003845	*fadB*	3-hydroxyacyl-CoA dehydrogenase	1.1.1.35 4.2.1.17 5.1.2.3 5.3.3.8
SCB32_GM000049	*fadN*	3-hydroxyacyl-CoA dehydrogenase	1.1.1.35
SCB32_GM004049	*fadA, fadI*	acetyl-CoA acyltransferase	2.3.1.16
SCB32_GM002132	*fadJ*	3-hydroxyacyl-CoA dehydrogenase	1.1.1.35 4.2.1.17 5.1.2.3
SCB32_GM005158	*GCDH, gcdH*	glutaryl-CoA dehydrogenase	1.3.8.6
SCB32_GM000044	*paaH, hbd, fadB, mmgB*	3-hydroxybutyryl-CoA dehydrogenase	1.1.1.157
SCB32_GM004897	*E2.3.1.9, atoB*	acetyl-CoA C-acetyltransferase	2.3.1.9
SCB32_GM001586	*E3.1.2.23*	4-hydroxybenzoyl-CoA thioesterase	3.1.2.23
SCB32_GM002139	*paaF, echA*	enoyl-CoA hydratase	4.2.1.17
SCB32_GM003170	*E3.5.1.4, amiE*	amidase	3.5.1.4
SCB32_GM001571	*acyP*	acylphosphatase	3.6.1.7
SCB32_GM000841	*ubiX, bsdB, PAD1*	flavin prenyltransferase	2.5.1.129

References

1. S.M. Ha, C.K. Kim, J. Roh, J.H. Byun, S.J. Yang, S.B. Choi, J. Chun, and D. Yong: “Application of the Whole Genome-Based Bacterial Identification System, TrueBac ID, Using Clinical Isolates That Were Not Identified With Three Matrix-Assisted Laser Desorption/Ionization Time-of-Flight Mass Spectrometry (MALDI-TOF MS) Systems.” *Annals of Laboratory Medicine.* vol. 39, no. 6, pp. 530–536, 2019.
2. B. Liu, D. Zheng, Q. Jin, L. Chen, and J. Yang: “VFDB 2019: a comparative pathogenomic platform with an interactive web interface.” *Nucleic Acids Research*. vol. 47, no. D1, pp. D687–D692, 2019.
